# Mitochondrial Calcium Uniporter Beta in Alzheimer’s Disease

**DOI:** 10.1002/alz.086190

**Published:** 2025-01-03

**Authors:** Sarah D Kaye, Darpan Raghav, Ashlesha Kadam, John W Elrod, Dhanendra Tomar, Pooja Jadiya

**Affiliations:** ^1^ Wake Forest University School of Medicine, Winston‐Salem, NC USA; ^2^ Temple University, Philadelphia, PA USA

## Abstract

**Background:**

Alzheimer’s disease (AD) is a complex neurodegenerative disorder marked by progressive memory loss and cognitive decline. The precise molecular mechanisms underlying AD pathogenesis remain uncertain, underscoring the need for further investigation to identify novel therapeutic targets. We recently demonstrated that mitochondrial calcium (_m_Ca^2+^) overload significantly contributes to the development of AD, capable of independently driving AD‐like pathology. Modulating _m_Ca^2+^ dynamics, such as blocking _m_Ca^2+^ uptake, holds promise in reducing neuronal cell death in AD. However, these approaches challenge bioenergetic processes due to the role _m_Ca^2+^ plays in metabolic regulation. Therefore, it is essential to identify a regulator that can maintain _m_Ca^2+^ homeostasis while preserving bioenergetic functions.

**Methods:**

To identify a new molecular regulatory mechanism for reducing _m_Ca^2+^ overload and preserving bioenergetics in AD, we focused on Mitochondrial Calcium Uniporter Beta (MCUB), a negative regulator of _m_Ca^2+^ uptake. Initially, we examined MCUB expression in AD patient samples, AD model mice, and AD cell lines. Observing reduced MCUB expression in AD, we used 5xFAD mutant mice with neuronal‐specific MCUB overexpression to assess age‐associated changes in cognitive function, neuropathology, and mitochondrial bioenergetics. A neuroblastoma cell line expressing human amyloid precursor protein with the Swedish mutation (N2a/APPswe) and adenovirus encoded MCUB was examined for alterations in mitochondrial Ca^2+^ dynamics and functions.

**Results:**

Our findings revealed that expression of MCUB is lost in non‐familial AD samples, AD mouse models, and AD cell lines. MCUB overexpression in 5xFAD mice rescued spatial and fear‐associated memory impairments and alleviated amyloid beta pathology at 9 months of age. Furthermore, MCUB overexpression mitigated _m_Ca^2+^ overload, preserved crucial mitochondrial bioenergetic functions including oxidative phosphorylation (OXPHOS) and ATP production, and prevented neuronal cell loss.

**Conclusions:**

This study demonstrates for the first time that modulating MCUB holds therapeutic promise in preserving bioenergetics and mitigating neuronal damage and AD progression. These findings have the potential to significantly advance our understanding of AD pathogenesis and pave the way for effective treatments for AD.

